# Determining Microvascular Obstruction and Infarct Size with Steady-State Free Precession Imaging Cardiac MRI

**DOI:** 10.1371/journal.pone.0119788

**Published:** 2015-03-20

**Authors:** Wolfgang Wuest, Michael Lell, Matthias May, Michael Scharf, Christian Schlundt, Stephan Achenbach, Michael Uder, Axel Schmid

**Affiliations:** 1 Radiological Institute, Friedrich-Alexander-University-Erlangen-Nuremberg, Erlangen, Germany; 2 Department of Internal Medicine II, Division of Cardiology, Friedrich-Alexander-University-Erlangen-Nuremberg, Erlangen, Germany; University Hospital of Würzburg, GERMANY

## Abstract

**Purpose:**

In cardiac MRI (cMRI) injection of contrast medium may be performed prior to the acquisition of cine steady-state free precession (SSFP) imaging to speed up the protocol and avoid delay before late Gadolinium enhancement (LGE) imaging. Aim of this study was to evaluate whether a condensed clinical protocol with contrast cine SSFP imaging is able to detect early microvascular obstruction (MO) and determine the infarct size compared to the findings of LGE inversion recovery sequences.

**Materials and Methods:**

The study complies with the Declaration of Helsinki and was performed following approval by the ethic committee of the University of Erlangen-Nuremberg. Written informed consent was obtained from every patient. 68 consecutive patients (14 females/54 males) with acute ST-elevation myocardial infarction (STEMI) treated by percutaneous coronary revascularization were included in this study. CMRI was performed 6.6±2 days after symptom onset and MO and infarct size in early contrast SSFP cine imaging were compared to LGE imaging.

**Results:**

MO was detected in 47/68 (69%) patients on cine SSFP and in 41/68 (60%) patients on LGE imaging. In 6 patients MO was found on cine SSFP imaging but was not detectable on LGE imaging. Infarct size on cine SSFP showed a strong agreement to LGE imaging (intraclass correlation coefficient [ICC] of 0.96 for enddiastolic, p<0.001 and 0.96 for endsystolic, p<0.001 respectively). Significant interobserver agreement was found measuring enddiastolic and endsystolic infarct size on cine SSFP imaging (p<0.01).

**Conclusions:**

In patients after STEMI infarct size and presence of MO can be detected with contrast cine SSFP imaging. This could be an option in patients who are limited in their ability to comply with the demands of a cMRI protocol.

## Introduction

Primary percutaneous coronary intervention (PCI) is the treatment of choice in acute myocardial infarction (AMI) to recover normal blood flow by recanalisation of the occluded artery to reduce the extent of myocardial necrosis [1–2]. However, due to continuing microvascular obstruction (MO) reperfusion of the ischemic myocardium is incomplete in up to 30% of patients despite restoring epicardial blood flow. MO is associated with extensive ventricular remodelling and represents an important prognostic factor for impaired recovery of left ventricular function and long term survival [3–8].

Because of its excellent contrast and good spatial resolution gadolinium enhanced cardiac MRI (cMRI) is considered as the reference standard for the detection and assessment of MO and infarct volume [9]. Apart from late enhancement (LGE) imaging, which is commonly used to identify MO, there are several other imaging techniques to visualize MO in the early course after contrast administration. They include first pass perfusion with single-shot saturation recovery gradient-echo pulse sequences or early enhancement imaging with 3D inversion recovery gradient-echo sequences. However in clinical routine examination time is critical. To shorten the study duration standard cine steady-state free precession (SSFP) MRI imaging can be obtained after injection of contrast medium without loss of accuracy for regional and global LV ventricular function [10].

Recently Raff et al. [11] validated the accuracy of contrast enhanced cine MR imaging in the measurement of MO. Based on the findings of Raff el al. the aim of this study was to evaluate the differences of contrast-enhanced cine SSFP imaging and LGE imaging in a typical clinical protocol in order to assess the value of contrast enhanced SSFP imaging to serve as a backup option if LGE imaging is not evaluable or in case of an early abortion of the MRI scan.

## Materials and Methods

### Patients

68 consecutive patients (14 females/54 males) who presented with a first acute ST elevation myocardial infarction (STEMI) according to electrocardiographic and enzymatic criteria were prospectively enrolled. The mean age of the patients was 63±11 years (range 44–88 years). All patients had undergone successful PCI within 12 hours of symptom onset resulting in a Thrombolysis In Myocardial Infarction (TIMI) flow grade 2 or 3. Patients with a history of previous coronary revascularization (i.e. PCI or coronary artery bypass surgery), previous myocardial infarction, hemodynamic instability, renal failure or contraindication to cMRI examination (pacemaker, metal fragments, implants, severe arrhythmias or claustrophobia) were excluded. Local institutional review board approval was obtained and all patients gave written informed consent.

### cMRI protocol

CMR imaging was performed on two 1.5-T MRI systems (Magnetom Avanto and Magnetom Espree, Siemens, Erlangen, Germany) with dedicated phased-array cardiac receiver coils. The patients were placed in a supine position, and images were acquired at end expiratory breath hold with electrocardiogram gating. Initially scout images were acquired to localize the short axis of the heart. To depict myocardial edema a T2 weighted triple-inversion fast-spin-echo sequence was employed in three long axis (2-chamber, 3-chamber, 4-chamber view) and three short axis views (apical, midventricular, basal).

As a modification of the standard imaging protocol in which functional cine imaging is performed before the administration of contrast agents, cine imaging was acquired after the administration of 0,2 mmol/kg of a gadolinium-based contrast agent (Gadovist, Bayer Healthcare, Leverkusen, Germany). Three long axis and contiguous short axis views covering the entire left ventricle from the base to the apex were acquired with a retrospectively ECG-gated segmented k-space balanced steady state free-precession pulse sequence (trueFISP, Siemens medical Solutions). Image parameters were as follows: in plane resolution 1.6x1.6 mm, slice thickness 6 mm, gap 2 mm, echo time 1.6 ms, repetition time 3.2 ms, flip angle 60°, matrix 256x156, temporal resolution 35–50 ms.

LGE was performed 10–20 minutes after administration of contrast agents with a segmented 2-dimensional inversion recovery gradient-echo (IR GE) pulse sequence, with slice positioning identical to the SSFP cine images. In-plane resolution was 1.6x1.3 mm with a slice thickness of 6mm (gap 2mm, echo time 3.22ms, repetition time 700ms, flip angle 25°, matrix 256x154, triggering to every other heart beat). The inversion time was determined by an inversion-time scout sequence and typically ranged from 230–300ms to null the signal of normal myocardium.

In order to rule out that MO findings could be at least partially related to the used SSFP imaging sequence 7 additional consecutive patients (7 males/0 females) with a mean age of 60±10 years (range 49–76 years) were included

In these patients an early IR GE slice was immediately performed after each early cine SSFP slice with a MO finding during the first ten minutes and late cine SSFP slices were added during late IR GE imaging in cases when MO was present on early SSFP or early IR GE imaging.

### cMRI analysis

cMRI analysis was performed by two observers (W.W. and S.A.) with more than 4 and 10 years experience in cMRI, respectively, who were blinded to all patient and other imaging data. The final value of infarct and MO size used was an average of the two readings.

A manual segmentation of the endo- and epicardial borders of the LV wall in the short axis SSFP images was done in the endsystolic and enddiastolic phase and the global mass and functional parameters were analysed by using dedicated software (ARGUS; Siemens Healthcare, Erlangen, Germany). The trabecular network and the papillary muscles were defined as part of the blood pool.

The following parameters were evaluated: ejection fraction (EF), enddiastolic volume (EDV), endsystolic volume (ESV), stroke volume (SV) and myocardial mass.

**Fig 1:**
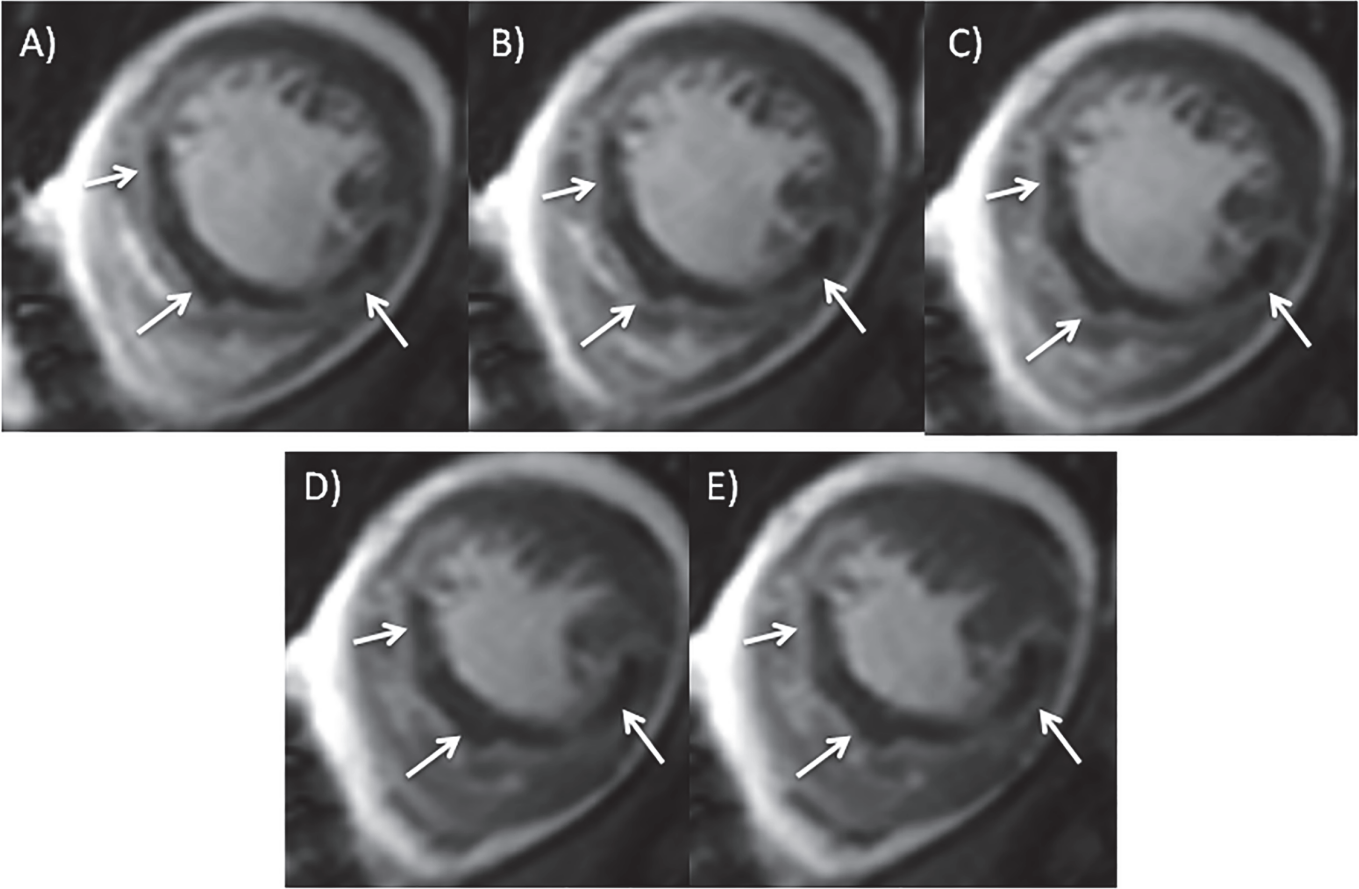
Typical MO appearance on early cine SSFP imaging at the same slice position during the cardiac cycle (A-E).

Infarcted tissue was defined as visually hyperenhanced myocardium in the inversion recovery sequence compared to the remote normal myocardium. MO was defined as mid- or subendocardial hypoenhancement within the hyperenhanced region and was considered as part of the infarct volume. To exclude low signal intensity artifacts in cine SSFP imaging potentially mimicking MO areas, a “true MO finding” was defined as a hypoenhancing myocardial area surrounded by a hyperenhancing infarction zone constantly visible located at the same position within the cardiac wall on each SSFP image during the complete cardiac cycle (see Fig. 1).

Infarct volume was measured on delayed enhancement imaging and on enddiastolic (ED) and endsystolic (ES) SSFP cine images by manually tracing the borders of the hyperenhanced areas on each slice. Infarct volume was measured in both, ED and ES cine SSFP imaging, to evaluate which imaging time point correlated better with LGE imaging.

Total infarct size was expressed as a percentage of global LV myocardial mass, given by the sum of all volumes of hyperenhanced regions for all slices multiplied by myocardial density (1.05 g/mL and divided through the global LV mass. The size of the MO areas was independently assessed on LGE images and SSFP cine images, by manually contouring the hypoenhanced areas within the infarcted myocardium. In SSFP images the whole cardiac cycle was evaluated regarding the presence of hypoenhancement within the infarcted myocardium. The total amount of MO was defined as the sum of MO areas for all slices multiplied by 1.05 g/mL and was expressed as a percentage of the total infarct size. To avoid bias, evaluation of delayed enhancement imaging and SSFP cine imaging was performed with a time delay of at least 4 weeks.

### Statistical analysis

Values were given as mean ± standard deviation. Agreements between cine SSFP and LGE imaging were determined by the intraclass correlation coefficient (ICC) and 95% confidence intervals. Bland-Altman plots were generated to graphically present the stability and consistency of the agreement between both tests [12,13]. Pearson correlation coefficient was used for MO and infarct size and to evaluate the influence of the time period between contrast media application and the occurrence of MO as well as the influence of the size of the MO area.

After testing for normal distribution and variances in Levene's statistic, a non parametric Wilcoxon two paired test was used for MO values and for myocardial function studies. Significance was assessed at a p-level of 0.05. Statistical analysis was performed using the software package SPSS Statistics, Version 19 (SPSS Inc./ IBM, Chicago, IL).

## Results

The infarct related artery was the left anterior descending artery in 29/68 (43%), the left circumflex artery in 11/68 (16%) patients and the right coronary artery in 28/68 (41%) patients.

CMR imaging was performed 6.6±2 days after onset of myocardial infarction symptoms on average. Short axis cine SSFP imaging started 2.7±2.2 min and was completed 7.8±2.5 min after administration of contrast agent. The average time interval between the injection of contrast medium and the slice on which MO was present on cine SSFP imaging was 4.6±1.6 min. LGE imaging was performed between 15.5±3 min and 21.6±3.7 min after administration of contrast agent. The average time interval between the injection of contrast medium and the detection of MO areas on late enhancement imaging was 17.3±2.5 min.

**Fig 2:**
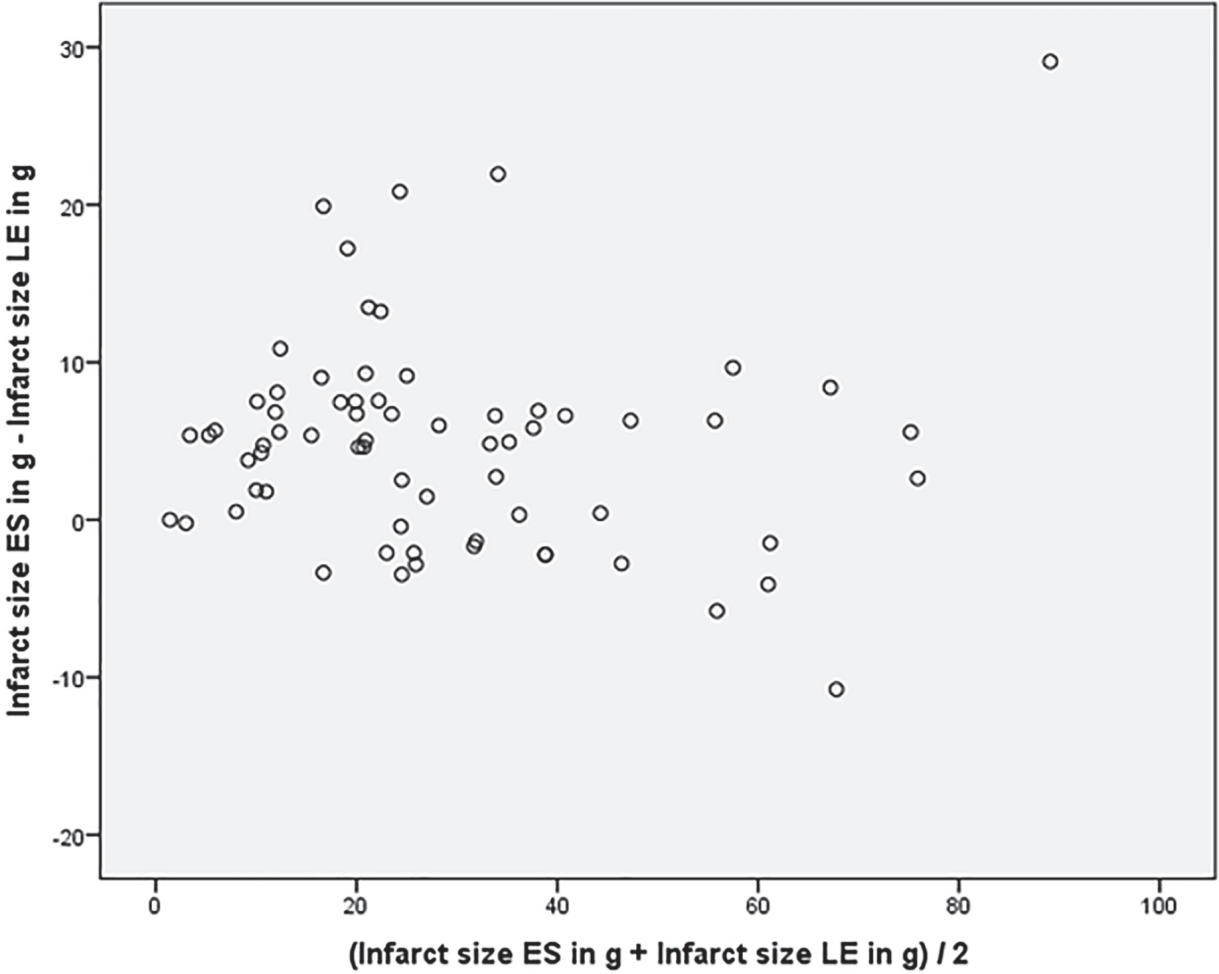
Bland-Altman plot for ES infarct size indicate that differences between the tests are consistent and the majority of them are within the 1.96 SD intervals.

**Fig 3:**
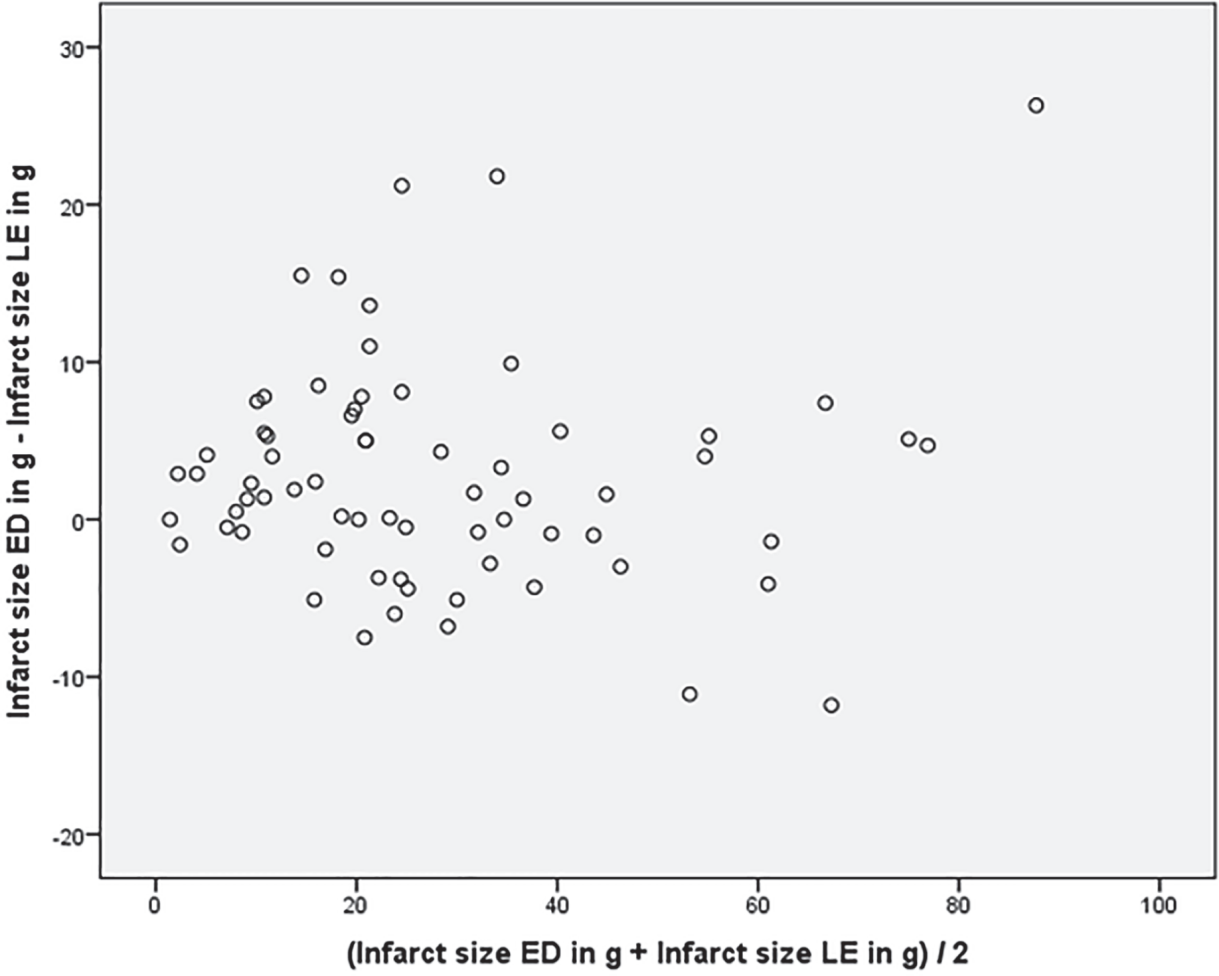
Bland-Altman plot for ED infarct size indicate that differences between the tests are consistent and the majority of them are within the 1.96 SD intervals.

In all 68 patients, areas of hyperenhancement were visible with LGE imaging as an indication of infarcted LV myocardium. The mean global extent of infarcted myocardium with LGE imaging was 27±20g, the mean total infarct size in correlation to LV mass was 18%±12%. Infarct size in ED and ES SSFP imaging was 29g±20g and 31g±20g respectively. The global extent of infarct size on ED and ES cine SSFP imaging showed a strong agreement to LGE imaging (ICC ES: 0.955, 95% CI: 0.844–0.981, p<0.001 and ICC for ED: 0.963, 95% CI: 0.932–0.979, p<0.001). Bland-Altman plots indicated that variability of the differences between the two tests (ED/ES cine SSFP vs. LGE imaging) for both measures was consistent and the majority of them were within the 1.96 SD intervals (see Figs. 2–3). Significant interobserver agreement was found measuring ED and ES infarct size (pearson correlation: r = 0.81 and r = 0.83, p<0.01).

MO was present on 41/68 (60%) patients on LGE imaging. Patients with MO on LGE imaging had a significantly larger infarct size compared to patients without MO (33.3±25.6g vs. 15.3±15.3g, p = 0.001) and larger enddiastolic left ventricular volume (LVEDV 164±32ml vs. 147±33ml, p = 0.05). The difference in global left ventricular function (LVEF 49±11% in patients with MO vs. 51±11% in patients without MO, p = 0.3) and left ventricular mass in patients with MO with LGE imaging (145±32g in patients with MO vs. 130±34g in patients without MO, p = 0.07) did not reach statistical significance.

**Fig 4:**
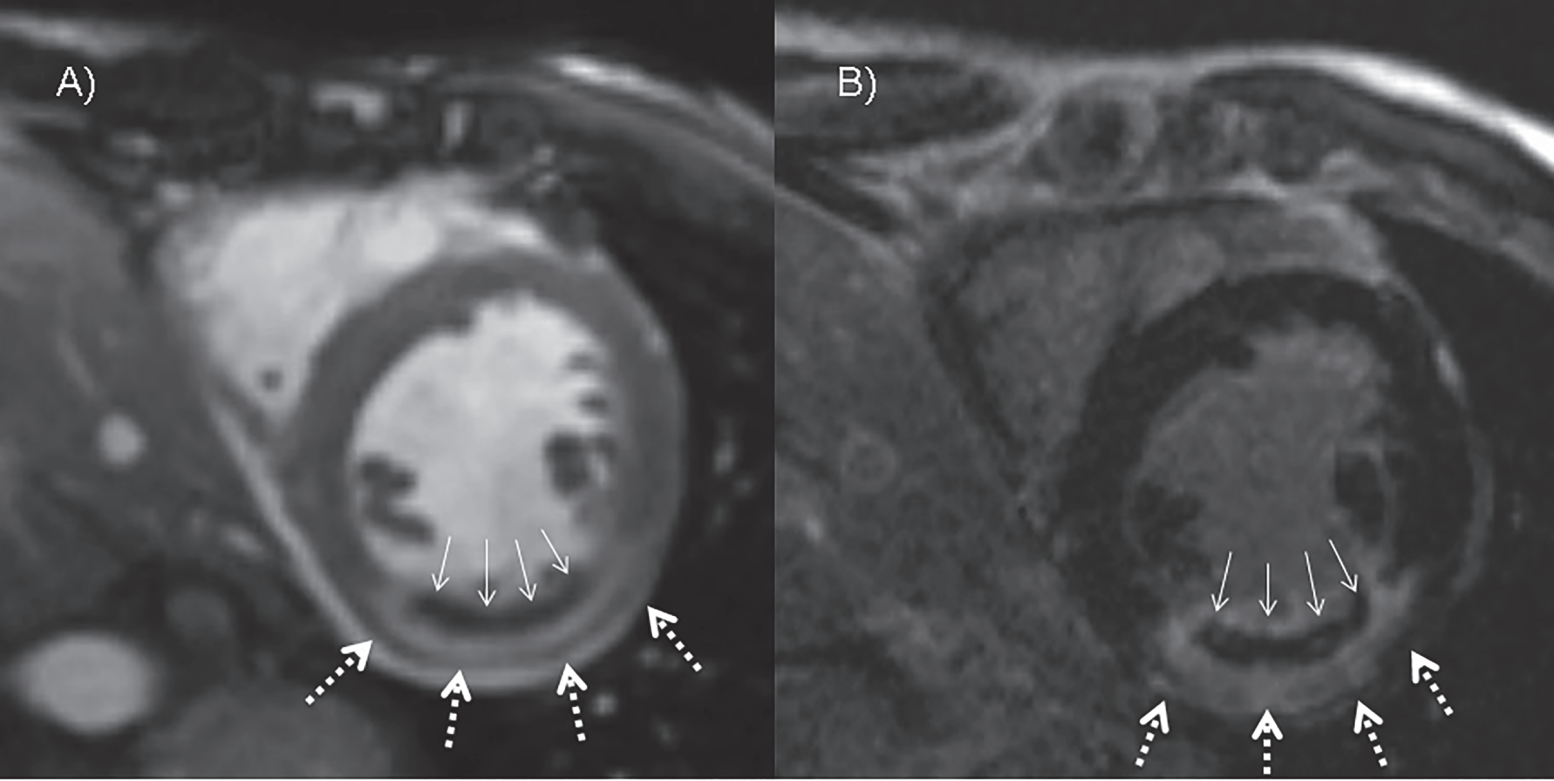
Cine and LGE imaging after 6 and 14.5 minutes respectively. Both images (A and B) show a well demarcated hyperintense midventricular inferolateral myocardium with a subendocardial located crescent shaped hypointensity representing myocardial infarction with MO (arrows: MO size, dotted arrows: infarct size).

**Fig 5:**
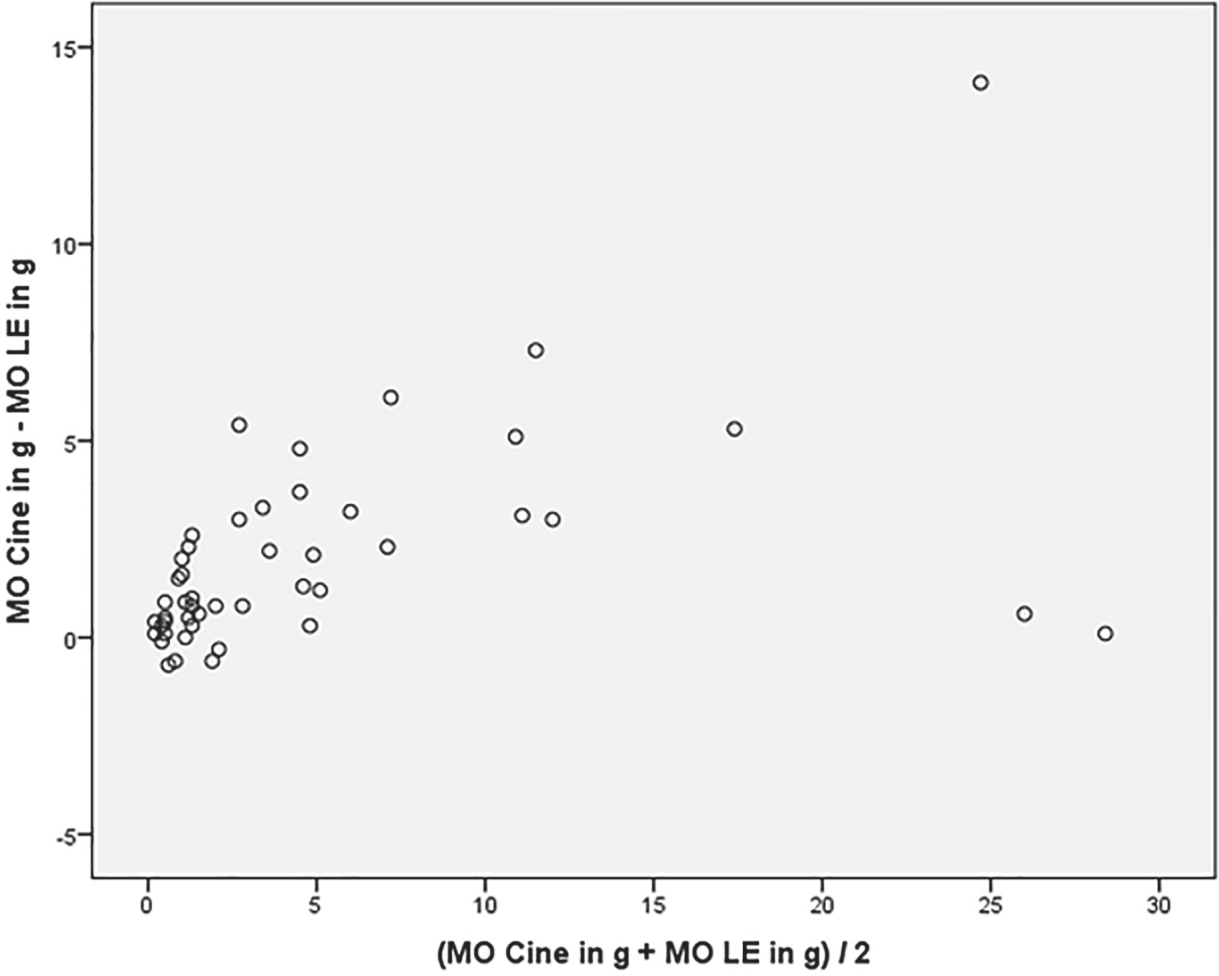
Bland-Altman plot for MO indicates that differences between the tests are consistent and the majority of them are within the 1.96 SD intervals.

On early cine SSFP imaging MO was identified in 47/68 (69%) patients. MO extent on cine imaging correlated significantly with infarct size (pearson correlation: r = 0.826, p<0.01) and with MO extent on LGE imaging (ICC of 0.946, 95% CI: 0.809–0.978, p<0.001) (see Fig. 4). Bland-Altman plot indicated that variability of the differences between the two tests was consistent and the majority of them were within the 1.96 SD intervals (see Fig. 5).

**Fig 6:**
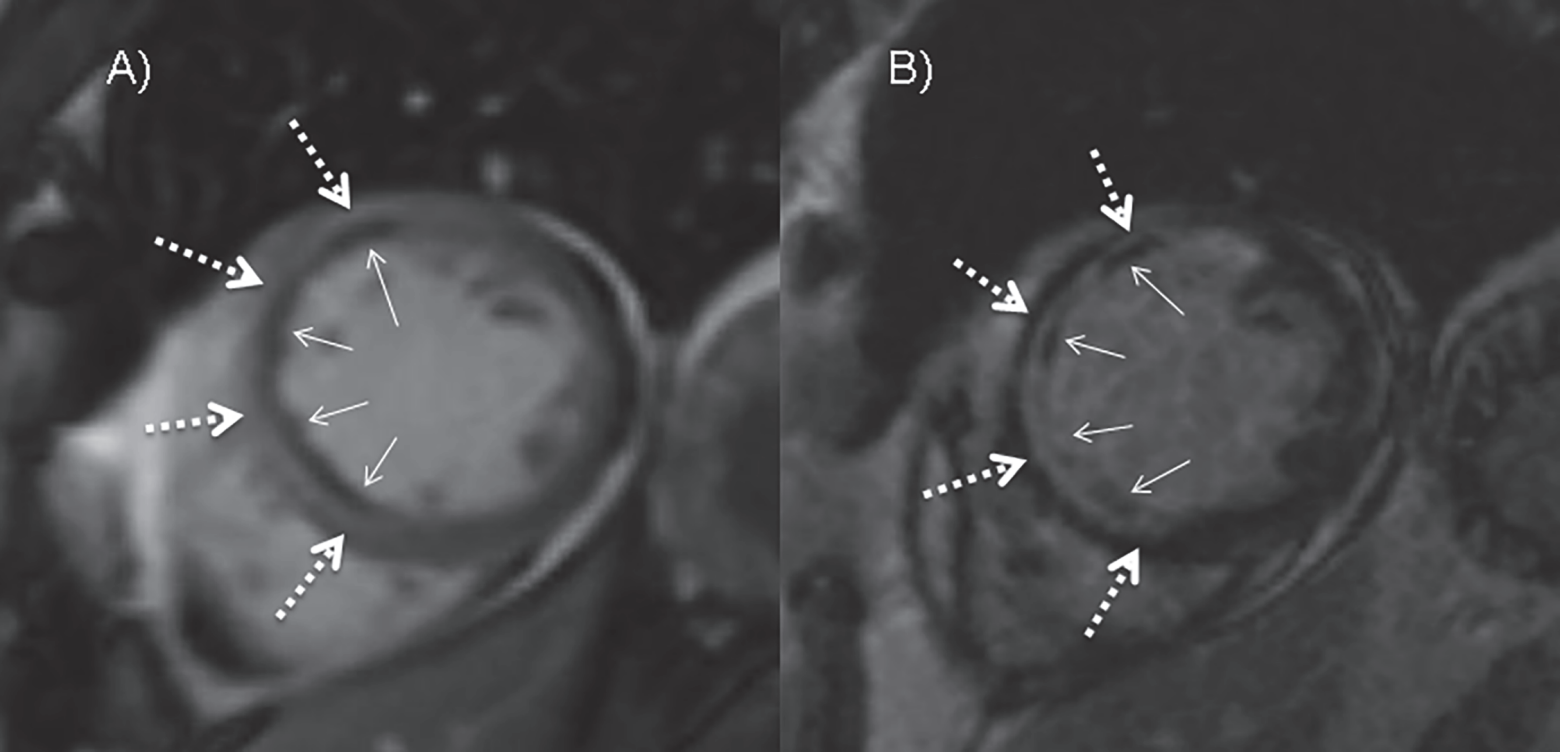
Comparison between MO on early cine SSFP and LGE. A) Cine imaging performed 4 minutes after contrast medium injection. Midventricular subendocardial MO reaching from septal, anteroseptal to anterior. B) LGE imaging 16.5 minutes after contrast medium injection. Hyperenhanced midventricular septal, anteroseptal to anterior myocardium with less MO compared to cine imaging (arrows: MO size, dotted arrows: infarct size).

The global extent of MO detected on early cine SSFP imaging and the MO size in correlation to infarct size and LV mass were significantly larger than those on LGE imaging (p<0.001, see Table 1) representing an early hypoenhancement—late hyperenhancement pattern (see Fig. 6). MO size on cine SSFP imaging correlated with the difference between MO on cine SSFP and LGE imaging (r = 0.623, p<0.01). MO size correlated inversely with the time point of occurrence on cine SSFP imaging without reaching significance (r = −0.28, p = 0.06). No significant correlation was found between the time point of the occurrence of MO on early cine SSFP imaging and the difference of MO size on cine SSFP imaging and LGE imaging. (r = −0.115, p = 0.44).

**Table 1:** MO/infarct size in patients with MO on SSFP cine and late enhancement imaging.

Sequence	MO global extent	MO size/infarct size	MO size/LV mass
SSFP	5.9±7.5g	15%±10%	4%±4%
LE	3.9±6.2g	9%±9%	2%±3%

**Fig 7:**
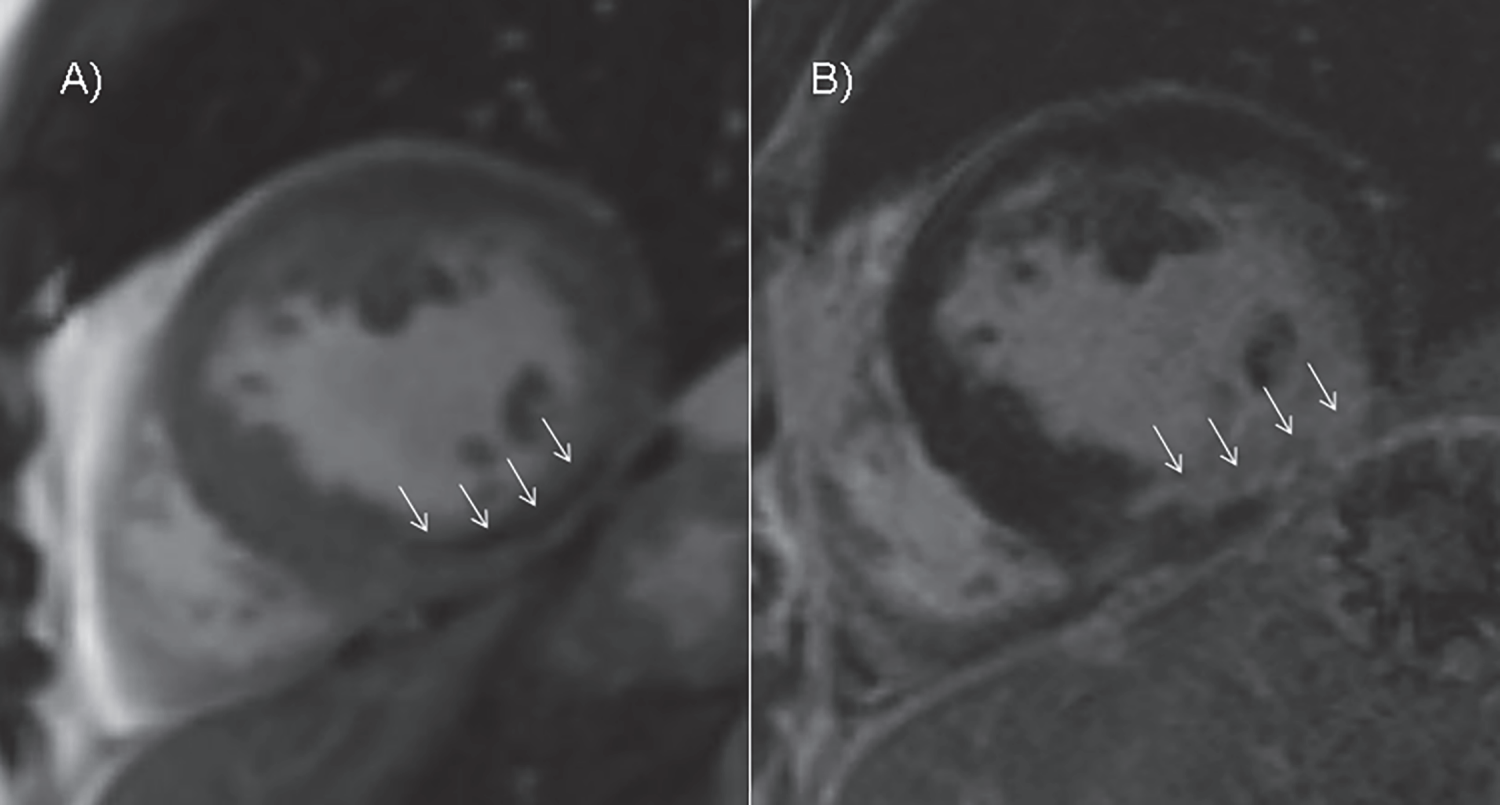
Comparison between MO on early cine SSFP and LGE. A) Cine imaging performed 4 minutes after contrast medium injection. A demarcated crescent shaped hypointensity representing MO in the midventricular inferior myocardium. B) LGE imaging 19 minutes after contrast medium injection. Hyperenhanced midventricular inferior myocardium missing MO at an identical slice position compared to cine imaging (see arrows for comparison).

All areas of MO detected on LGE imaging were also demonstrated with early cine SSFP. In 6 patients MO was found on cine SSFP imaging but was not present on LGE imaging (see Fig. 7). In these patients the size of the MO area in cine SSFP was 2.2±1.5g vs. 6.4±7.8g when MO was seen in both sequences (see Table 2). The infarct size of patients with MO only visible on cine SSFP imaging was lower than in patients with MO on both imaging methods (26±17g, 17% ± 8% of LV mass vs. 34±26g, 23% ± 16% of LV mass).

**Table 2:** MO/ infarct size in patients with MO on cine imaging not detectable on late enhancement imaging compared to patients with MO on both SSFP and LE imaging.

Sequence	MO size/infarct size	MO size/LV mass
SSFP	10%±6%	1%±1%
SSFP+LE	16%±11%	4%±4%

**Fig 8:**
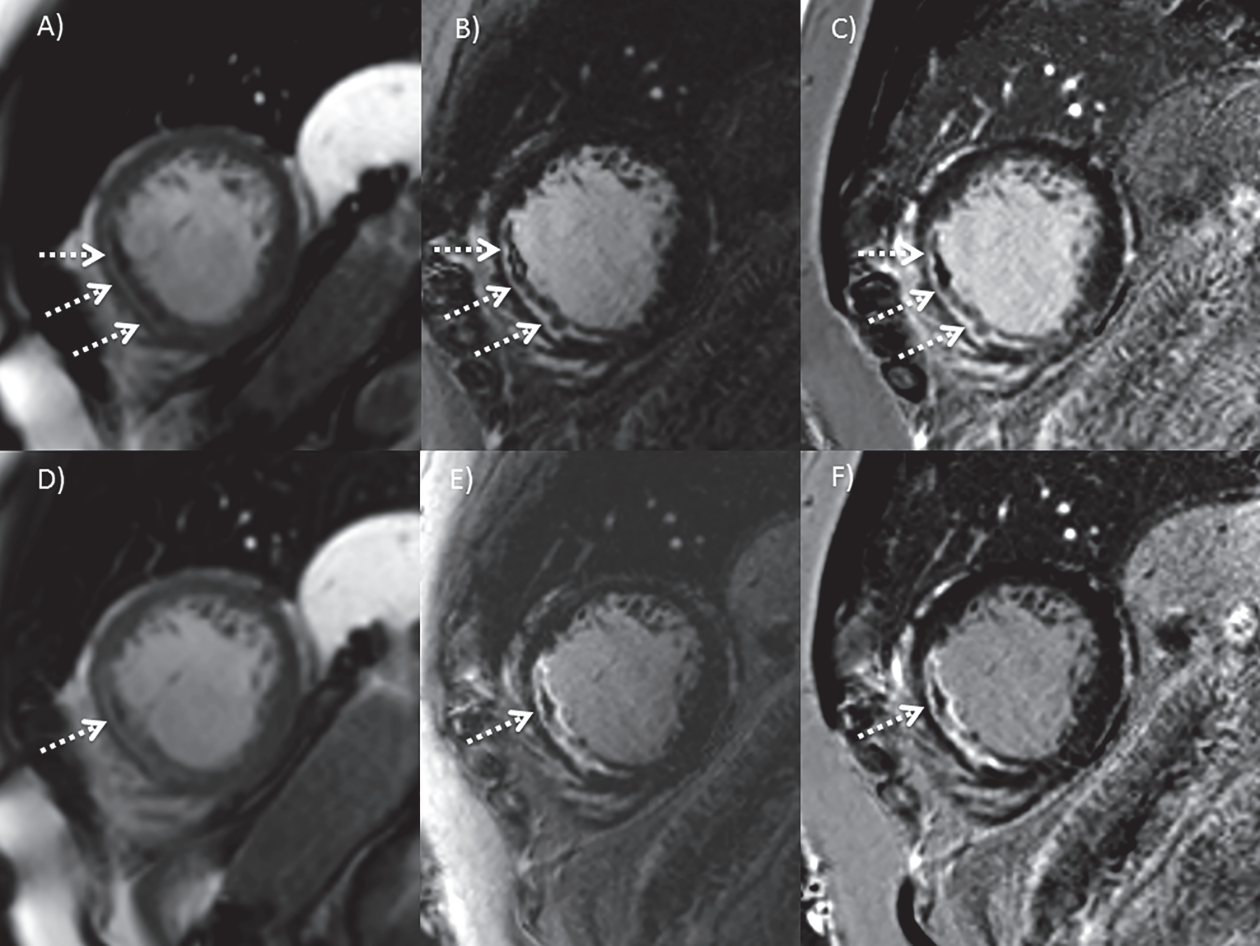
MO with different imaging methods. Early enhancement 9 min after contrast agent: A) Cine SSFP B) IR GE imaging and C) PSIR. Late enhancement 23 min after contrast agent: D) Cine SSFP E) IR GE imaging and F) PSIR. Note the comparable extent of MO with SSFP and IR imaging at each time point, but the greater extent of MO at early imaging (A-C) compared to late imaging (D-F).

In the 7 additionally scanned patients MO was present in 5/7 cases. In all 5 cases, the extent of MO was equal on early SSFP imaging compared to early IR GE imaging as well as on late SSFP imaging compared to late IR GE imaging (see Fig. 8). The extent of MO decreased uniformly on both sequences between early and late imaging.

## Discussion

Our study demonstrates that SSFP cine imaging performed after contrast administration allows for detection of infarct size and microvascular obstruction in close agreement to LGE imaging.

Infarct size represents an important predictor for left ventricular remodeling which could progress to heart failure and is associated with increased morbidity and mortality [14]. In our study both enddiastolic and endsystolic steady-state free precession imaging allowed for determining infarct size with a strong agreement with the gold standard LGE imaging. We found a significant positive correlation between infarct size and MO as it has been described for other MRI techniques before [15–18].

Apart from infarct size, MO is an important predictor of heart failure after myocardial infarction. If present, MO is associated with poor functional recovery and myocardial remodeling [19–20] and represents an independent prognostic factor for adverse outcomes after acute myocardial infarction [5,19,21]. MO is caused by compression of intramyocardial vessels by intracellular edema [15,22], hypoxic endothelial damage [13], thromboembolic obstruction [7] and regional spasm of small intramyocardial vessels [23] in the acute and subacute phase of myocardial infarction. Angiography based techniques such as the corrected TIMI frame count or myocardial blush grade are lacking sensitivity and specificity for the assessment of MO since they do not allow direct visualization of perfusion at the level of the myocardial microvascular system [16,24]. Moreover, the extent of MO has been shown to increase within the first 2 days after myocardial infarction and, therefore, the total extent of MO may be underestimated if assessed immediately after reperfusion [25]. In contrast, cMRI is an ideal, non-invasive imaging modality, since it combines the highly accurate assessment of morphological and functional parameters with the delineation of tissue necrosis and MO. Hypoenhancement within an area of hyperenhancing infarcted myocardium has been proven to represent persistent limited myocardial perfusion within the infarct core despite restoration of coronary blood flow after reperfusion [25,26]. These hypoenhancing defects within the infarction are verifiable only in the course of acute and subacute infarction and disappear at later stages of chronic infarction [26].

However, studies on the incidence of MO differ in terms of the MRI sequence and the time delay at which MO has been assessed after the administration of contrast agents. While enhancement defects within the infarcted myocardium persisting for more than 10 minutes after contrast administration (late MO) have been studied on inversion recovery gradient-echo sequences designed for late enhancement imaging, the early course of perfusion defects within the first minutes after contrast administration has traditionally been assessed either by first-pass perfusion techniques or by 2D or 3D gradient-echo sequences (early MO). Some studies postulate that detection of MO in early enhancement leads to a higher risk for upcoming major adverse cardiac events (cardiac death, reinfarct, rehospitalisation due to heart failure, unstable angina [5,23], some do not [21]. Bekkers et al. [27] studied 84 consecutive patients with a reperfused AMI 5 and 104 days after admission using a single breath-hold 3D inversion recovery gradient echo (IR-GRE) pulse sequence. In 8 patients (10%) MO disappeared on LGE compared to early enhancement. These patients had an LVEF that was intermediate at baseline and after follow-up. Bekkers et al. speculated that these patients represent an intermediate risk group and should not be excluded from future therapies for MO.

The technique of contrast enhanced cine SSFP imaging as it has been performed in our study has been proposed only once before in a study by Raff et al. [11]. They validated the accuracy of contrast enhanced cine MR imaging in the measurement of MO by comparing hypoenhancing defect sizes between a standard cine SSFP sequence obtained after the administration of contrast material and a first-pass perfusion gradient-echo sequence in 80 patients and an early inversion recovery gradient-echo sequence in 50 patients. Contrast enhanced cine SSFP imaging was performed immediately after first-pass perfusion imaging and the early inversion-recovery section, prepared by a preliminary inversion-time scout sequence, was obtained immediately after each contrast-enhanced cine section. No number is stated how many patients had early MO but late MO disappearance but they found early hypoenhancement-late hyperenhancement in all of their 18 patients, who underwent repeat contrast enhanced cine MRI after 10 and 30 minutes. Findings at contrast-enhanced cine MR imaging agreed with the global and transmural extent of microvascular obstruction at first-pass perfusion and inversion-recovery gradient-echo MR imaging. The authors concluded that cine SSFP imaging provides data on MO with results that compare very closely to standard MR imaging techniques. However, in a typical condensed clinical protocol, contrast enhanced cine MR imaging is obtained within the first minutes after the administration of contrast media, whereas late-enhancement imaging is obtained 10–20 minutes after the application of contrast media. Therefore, even if comparable at approximately the same imaging time points as shown by Raff et al., the results of early contrast enhanced cine imaging and late enhancement imaging might differ due to the influence of an early hypo-/late hyperenhancement pattern [15,23]. Based on the findings of Raff el al. the aim of this study was to evaluate the differences of contrast-enhanced cine SSFP imaging and LGE imaging in a typical clinical protocol in order to assess the value of contrast enhanced SSFP imaging to serve as a backup option if LGE imaging is not evaluable or in case of an early abortion of the MRI scan. Therefore, in contrast to Raff et al., besides MO size also infarct size was measured on ED and ES cine SSFP imaging and was evaluated for correlation with LGE imaging. We found a good correlation between both, the size of the infarcted area as well as the size of the MO area on cine SSFP imaging and LGE imaging but a slightly greater extent of the overall MO area on early cine SSFP imaging compared to LE imaging. The greater extent of MO can be explained by the earlier imaging time points on cine SSFP compared to LGE imaging and is not related to the used imaging sequence as shown in the 5 additionally scanned patients. To draw a conclusion from the size of MO for therapy planning it is important to be aware of the imaging time point since early imaging slightly overestimates MO. Generally, this could be explained by the effects of slow passive diffusion of contrast media into the infarcted zone resulting in an early hypo-/ late hyperenhancement pattern as described before by others [28–29]. The concept of a slow passive diffusion process of contrast media into the infarcted area might serve also as an explanation for the fact that in a certain number of patients (6/47) with MO on SSFP imaging MO areas could be detected exclusively on early cine SSFP imaging without any hypoenhancing infarction core on LGE imaging.

While the median MO size (2g) in these patients was small compared to the overall median MO size (6g), small MO areas did not disappear in all cases between early and late enhancement imaging, indicating different diffusion properties in different patients. We can only speculate but this could be due to different diffusion coefficients or pathological permeability changes.

Since MO has been described as a negative predictor for the clinical outcome, our data is raising the question, whether this constellation of findings is representing a group of patients on an intermediate risk which, however, certainly warrants larger follow-up studies. Furthermore, the clinical significance of a virtually absent decrease of MO area on LE imaging as observed in some patients with even larger MO extent remains unclear but could also be of potential interest for further investigations.

### Limitations

Some limitations need to be stated. First, the study was not designed as a longitudinal follow up of the functional recovery or the clinical outcome of the patients. Therefore it is not possible to draw any conclusions regarding prognosis particularly in the subgroup of patients with MO on cine SSFP imaging not found in LGE imaging. Secondly, the study population was rather small and—even though only STEMI patients were included into the study—relatively inhomogenous regarding infarct and MO size. Against this background, further subgroup analysis with respect to clinical outcome appears not to be reasonable.

Third, the study protocol was designed as a condensed protocol consisting of SSFP and IR-GRE sequences. Therefore, we did not compare post contrast cine SSFP imaging to other sequences than IR-GRE.

Fourth, manual delineation was used to assess infarct size on LGE and SSFP images. An alternative technique is for example the use of a signal threshold (n-SD technique or full width half maximum) based on remote myocardium signal analysis. However at this time there is no dedicated statement of the “Task Force” regarding the optimal method for quantitative assessment [30].

Finally the time point of the occurrence of an infarction or MO area on cine SSFP imaging (roughly between 3 and 8 minutes post contrast administration) might represent a possible bias in our results. Since SSFP imaging has constantly been performed from the base towards the apex, more basal located MO areas might be overestimated compared to more apical located MO areas due to the difference in acquisition time point. While larger MO areas showed significantly more decrease in size between CE cine SSFP imaging and LGE imaging compared to smaller MO areas, no significant difference in the decrease in size of an “early” MO at a more basal slice compared to a “late” MO on a more apical slice was obtained in our study cohort.

In summary, even if MO size is slightly overestimated on contrast enhanced cine SSFP imaging in particular in the case of larger MO areas due to the earlier imaging time point, contrast enhanced cine SSFP imaging was able to detect all cases with MO on LGE imaging and could therefore indeed serve as a back-up if LGE imaging should not be evaluable.

## Conclusion

In patients after STEMI infarct size and presence of MO can be detected with early contrast enhanced cine SSFP imaging. This could be an option in patients who are limited in their ability to comply with the demands of a cMRI protocol.
